# Brown fat activation reduces hypercholesterolaemia and protects from atherosclerosis development

**DOI:** 10.1038/ncomms7356

**Published:** 2015-03-10

**Authors:** Jimmy F. P. Berbée, Mariëtte R Boon, P. Padmini S. J. Khedoe, Alexander Bartelt, Christian Schlein, Anna Worthmann, Sander Kooijman, Geerte Hoeke, Isabel M. Mol, Clara John, Caroline Jung, Nadia Vazirpanah, Linda P.J. Brouwers, Philip L.S.M. Gordts, Jeffrey D. Esko, Pieter S. Hiemstra, Louis M. Havekes, Ludger Scheja, Joerg Heeren, Patrick C.N. Rensen

**Affiliations:** 1 Division of Endocrinology, Department of Medicine, Leiden University Medical Center, Albinusdreef 2, Leiden 2333 ZA, The Netherlands; 2 Einthoven Laboratory for Experimental Vascular Medicine, Leiden University Medical Center, Albinusdreef 2, Leiden 2333 ZA, The Netherlands; 3 Department of Pulmonology, Leiden University Medical Center, Albinusdreef 2, Leiden 2333 ZA, The Netherlands; 4 Department of Biochemistry and Molecular Cell Biology, University Medical Center Hamburg-Eppendorf, Martinistraße 52, Hamburg 20246, Germany; 5 Department of Genetics and Complex Diseases, Harvard School of Public Health, Boston, Massachusetts 02115, USA; 6 Diagnostic and Interventional Radiology, University Medical Center Hamburg-Eppendorf, Martinistraße 52, Hamburg 20246, Germany; 7 Department of Cellular and Molecular Medicine, University of California–San Diego, La Jolla, California 92093, USA; 8 Department of Cardiology, Leiden University Medical Center, Albinusdreef 2, Leiden 2333 ZA, The Netherlands; 9 Netherlands Organization for Applied Scientific Research—Metabolic Health Research, Gaubius Laboratory, Zernikedreef 9, Leiden 2333 CK, The Netherlands

## Abstract

Brown adipose tissue (BAT) combusts high amounts of fatty acids, thereby lowering plasma triglyceride levels and reducing obesity. However, the precise role of BAT in plasma cholesterol metabolism and atherosclerosis development remains unclear. Here we show that BAT activation by β3-adrenergic receptor stimulation protects from atherosclerosis in hyperlipidemic APOE*3-Leiden.CETP mice, a well-established model for human-like lipoprotein metabolism that unlike hyperlipidemic *Apoe*^−/−^ and *Ldlr*^−/−^ mice expresses functional apoE and LDLR. BAT activation increases energy expenditure and decreases plasma triglyceride and cholesterol levels. Mechanistically, we demonstrate that BAT activation enhances the selective uptake of fatty acids from triglyceride-rich lipoproteins into BAT, subsequently accelerating the hepatic clearance of the cholesterol-enriched remnants. These effects depend on a functional hepatic apoE-LDLR clearance pathway as BAT activation in *Apoe*^−/−^ and *Ldlr*^−/−^ mice does not attenuate hypercholesterolaemia and atherosclerosis. We conclude that activation of BAT is a powerful therapeutic avenue to ameliorate hyperlipidaemia and protect from atherosclerosis.

## Abstract (web summary)

Brown adipose tissue (BAT) produces heat by burning lipid triglycerides. Here, Berbée *et al*. show that pharmacological BAT activation protects hyperlipidemic mice from atherosclerosis, provided mice retain the metabolic capacity to clear cholesterol-enriched lipoprotein remnants by the liver.

## Graphical abstract

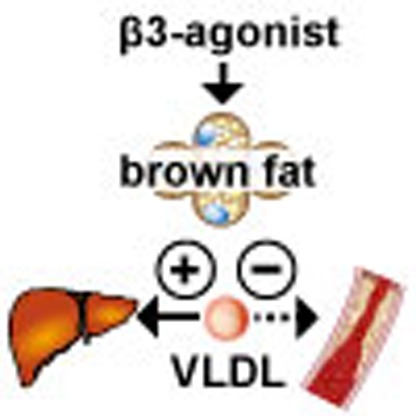

Cardiovascular diseases are the leading cause of morbidity and mortality in the Western world. Hyperlipidaemia, and in particular hypercholesterolaemia, is a major risk factor for the development of atherosclerosis, the main underlying cause of cardiovascular diseases. As current treatment strategies of atherogenic hyperlipidaemia prevent only 30% of all cardiovascular events^1^, novel treatment strategies are highly warranted.

Brown adipose tissue (BAT) is a highly active metabolic tissue that is present and active in adults^2^,^3^,^4^,^5^,^6^,^7^. Brown adipocytes not only reside in BAT depots but also lie scattered in certain white adipose tissue (WAT) depots. The development of these so-called peripheral, inducible brown adipocytes or ‘beige/bright cells’ is termed ‘browning’^8^,^9^. Both brown and beige adipocytes are functionally thermogenic and characterized by a large number of mitochondria and numerous small lipid droplets^10^. Physiologically, cold exposure activates BAT via stimulation of noradrenalin release by sympathetic neurons, which subsequently binds to β3-adrenergic receptor (β3-AR) on the brown adipocyte membrane^11^. As the β3-AR is selectively found on brown and white adipocytes, and WAT does not substantially contribute to energy expenditure (EE), the cold-stimulated activation of brown adipocytes inducing thermogenesis can be pharmacologically mimicked by selective β3-AR agonists such as CL316243, one the most selective β3-AR agonists available^11^,^12^. However, cold exposure induces systemic hormonal changes and ultimately leads to marked adipose tissue remodelling, including proliferation and increased BAT mass. β3-AR agonists, in contrast, along with inducing browning are only activating existing BAT^11^. As in cold exposure shivering thermogenesis is modulated and many other hormonal changes occur, β3-AR agonists represent an excellent experimental tool to study the effects of BAT activation selectively. Activation of the β3-AR on brown adipocytes rapidly induces intracellular lipolysis of triglycerides (TGs) from lipid droplets, resulting in release of fatty acids (FAs) into the cytoplasm. FAs are directed towards mitochondria where they either activate the uncoupling protein-1 (UCP1) in the inner membranes of mitochondria^13^ or undergo oxidation. The intracellular TG stores of brown adipocytes are rapidly replenished mainly by uptake of FA derived from lipolysis of TG-rich lipoproteins (TRLs) in the plasma^14^.

We showed that activation of BAT in mice via cold exposure^15^,^16^ or metformin treatment^17^ potently reduces plasma TG levels and obesity. Therefore, activation of BAT is now considered a promising new therapeutic avenue to combat hypertriglyceridemia and obesity^18^,^19^. However, increased lipolytic processing of plasma TRL naturally accelerates formation of pro-atherogenic cholesterol-rich remnants as well, which are usually cleared by the liver. Thus, Dong *et al*.^20^ described that BAT activation by cold exposure aggravates hypercholesterolaemia and atherosclerosis development in *Apoe*^−/−^ and *Ldlr*^−/−^ mice, which are the most widely used atherosclerosis mouse models. Apolipoprotein E (ApoE) is an apolipoprotein mediating the clearance of remnant lipoproteins through interacting with hepatic lipoprotein receptors, including the low-density lipoprotein receptor (LDLR)^21^. Although *Apoe*^−/−^ mice predominantly display an accumulation of cholesterol-enriched very-low-density lipoprotein (VLDL) remnants and very low high-density lipoprotein (HDL) levels^22^,^23^, *Ldlr*^−/−^ mice are characterized by high levels of remnant lipoproteins and LDL-cholesterol (LDL-C) without significant changes in HDL levels^24^. It is likely to be that the enhanced clearance of plasma TGs on BAT activation may require efficient clearance of cholesterol-enriched lipoprotein remnants by the liver, a pathway that is considered to be crucially dependent on a functional apoE-LDLR axis^21^.

The APOE*3-Leiden.CETP (*E3L.CETP*) model is a well-established model for hyperlipidaemia and atherosclerosis, which, unlike *Apoe*^−/−^ and *Ldlr*^−/−^ mice, responds well to the lipid-lowering and anti-atherogenic effects of statins^25^, fibrates^26^ and niacin^27^. *E3L.CETP* mice express a naturally occurring mutant form of human apoE3 that slows down remnant clearance, but does not completely abrogate the interaction with the LDLR^28^. This results in attenuated hepatic remnant clearance that is sufficient to induce hyperlipidaemia and atherosclerosis when feeding a Western-type diet (WTD), but, importantly, the hepatic remnant clearance route is still functional and can be modulated. In addition, *E3L.CETP* mice are transgenic for human cholesteryl ester transfer protein (CETP), which transfers cholesteryl esters from HDL to (V)LDL particles and for which rodents are naturally deficient. Hence, *E3L.CETP* mice are considered to display a more human-like lipoprotein metabolism.

Here we investigate the effects of β3-AR-mediated BAT activation^12^ on cholesterol metabolism and atherosclerosis development in *E3L.CETP* mice. Using TRL-mimicking particles, we show that the lipolytic conversion of TRLs by BAT increases the hepatic clearance of cholesterol-enriched lipoprotein remnants. This sequence of events results in a pronounced reduction in plasma TG and cholesterol levels, and ultimately in a marked attenuation of atherosclerosis development. In addition, we show that *Apoe*^−/−^ and *Ldlr*^−/−^ mice do not respond to the plasma cholesterol-lowering activity of BAT and are not protected from atherosclerosis development, underlining the importance of the apoE–LDLR axis for the anti-atherogenic activity of BAT.

## Results

### Activation of BAT augments FA combustion

**Figure 1:**
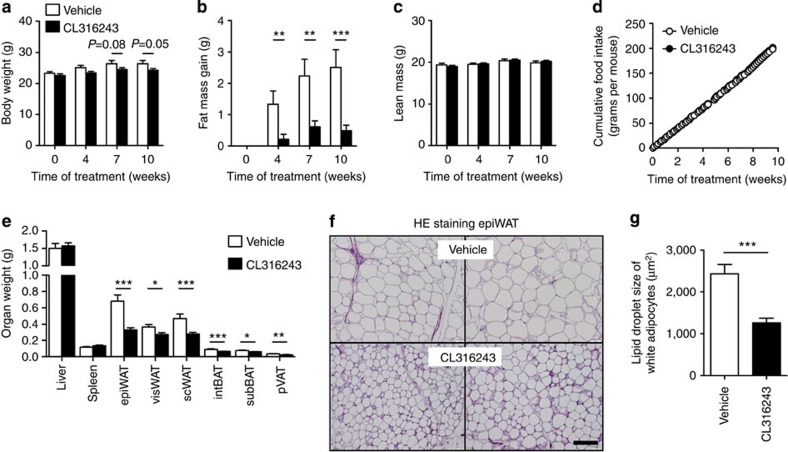
β3-AR agonism reduces body fat mass in *E3L.CETP* mice. (**a**) Body weight, (**b**) gain of total body fat mass, (**c**) lean mass and (**d**) cumulative food intake were determined in WTD-fed *E3L.CETP* mice at the indicated time points during treatment with the β3-AR agonist CL316243 or vehicle. (**e**) After necropsy at week 10, the weight of various organs was determined. (**f**) Epididymal WAT (epiWAT) was stained with haematoxylin–eosin (HE) and representative pictures are shown. Scale bar, 100 μm. (**g**) Lipid droplet size of white adipocytes was quantified. visWAT, visceral WAT; scWAT, subcutaneous WAT; intBAT, interscapular brown adipose tissue; subBAT, subscapular BAT; pVAT, perivascular adipose tissue. Values are means±s.e.m. (**a**–**e**: *n*=13–19 per group; **f**,**g**: *n*=8 per group). *P<0.05, **P<0.01, ***P<0.001 (unpaired two-tailed Student’s *t*-test).

We first assessed the effect of BAT activation on EE and fat storage in female *E3L.CETP* mice fed an atherogenic WTD for 10 weeks, while being treated with the selective β3-AR agonist CL316243 (3 × 20 μg per mouse per week; subcutaneous) or vehicle (phosphate-buffered saline (PBS)). β3-AR agonism tended to reduce body mass (up to −8%; Fig. 1a) and prevented total fat mass gain (up to −81%; Fig. 1b), without affecting lean mass (Fig. 1c) and cumulative food intake during the study (Fig. 1d). Accordingly, in the β3-AR agonist-treated mice the weight of the individual WAT pads was lower (ranging from −25% to −52%; Fig. 1e) and the average size of the lipid droplet within white adipocytes was smaller (−48%; Fig. 1f,g).

**Figure 2:**
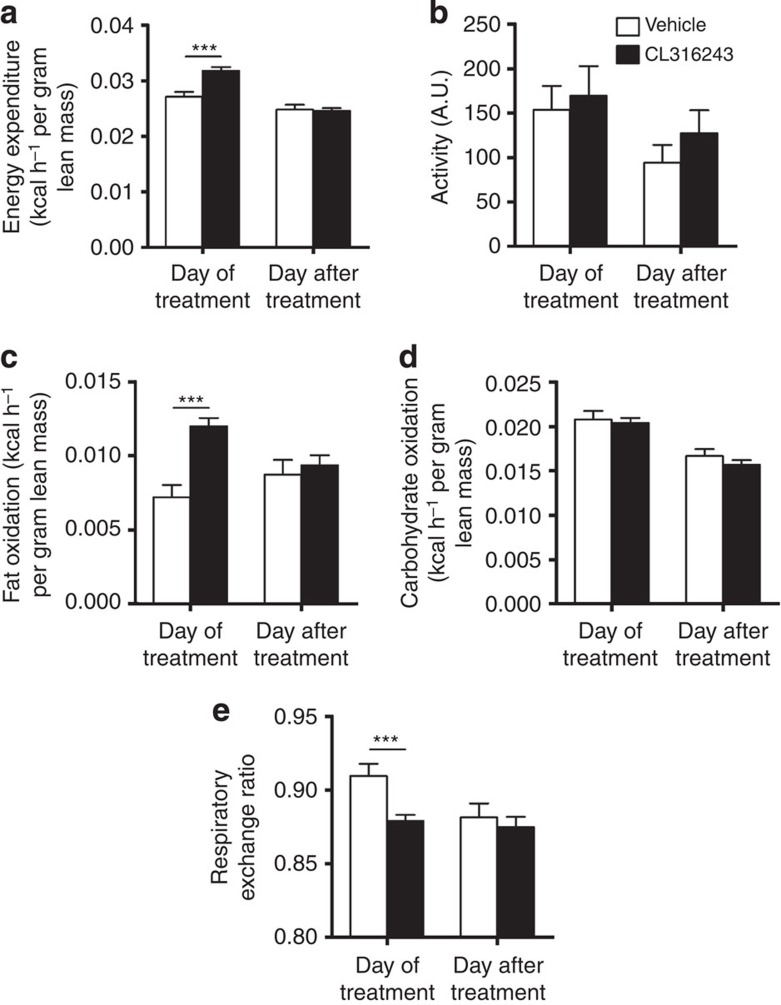
β3-AR agonism increases EE in *E3L.CETP* mice. (**a**) Mice were housed in fully automated metabolic cages and EE was determined and corrected for lean body mass. (**b**) Physical activity was monitored. In addition, (**c**) fat oxidation and (**d**) carbohydrate oxidation were determined and corrected for lean body mass. (**e**) Respiratory exchange ratio was determined. Data are shown as the first 12 h directly after the injection with CL316243 or vehicle (‘Day of treatment’) and the same 12-h period 24 h later (‘Day after treatment’). Values are means±s.e.m. (*n*=13–19 per group). ***P<0.001 (unpaired two-tailed Student’s *t*-test).

**Figure 3:**
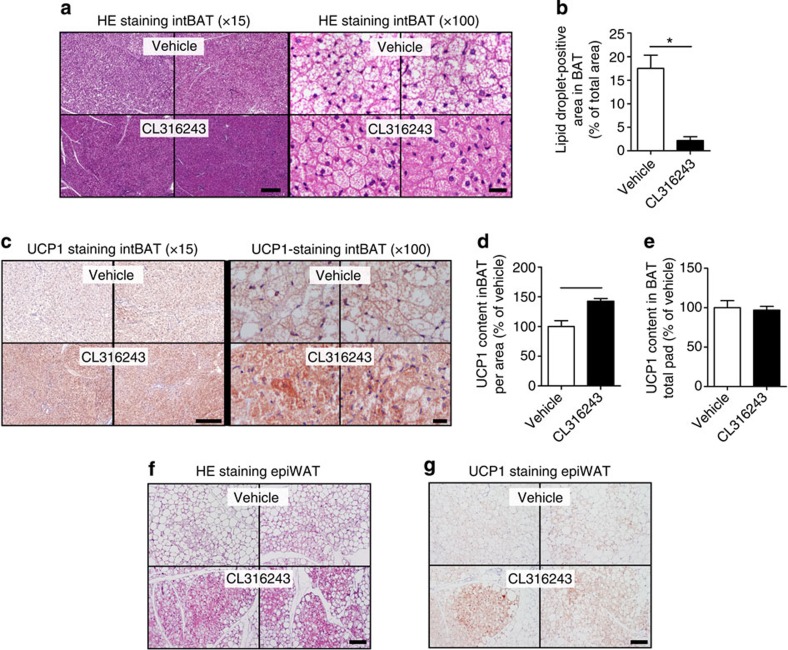
β3-AR agonism activates BAT and increases browning of WAT in *E3L.CETP* mice. Slides of intBAT of β3-AR agonist CL316243- and vehicle-treated *E3L.CETP* mice were (**a**) stained with haematoxylin–eosin (HE) or (**c**) immunohistochemically stained for UCP1, and representative pictures are shown. Scale bar, 200 μm (for × 15 original magnification) and 20 μm (for × 100 original magnification). (**b**) Lipid droplet-positive area, (**d**) UCP1 content per area and (**e**) UCP1 content of the total fat pad were quantified. Slides of epidydimal WAT (epiWAT) were stained with (**f**) HE and (**g**) immunohistochemically stained for UCP1, and representative pictures are shown. Scale bar, 100 μm. Values are means±s.e.m. (*n*=8 per group). *P<0.05, **P<0.01 (unpaired two-tailed Student’s *t*-test).

The β3-AR-mediated prevention of body fat gain was probably the consequence of increased adaptive thermogenesis, as total EE was markedly increased on the day of treatment (+17%; Fig. 2a) without differences in activity levels (Fig. 2b). The increase in EE was confined to increased FA oxidation (+67%; Fig. 2c) rather than carbohydrate oxidation (Fig. 2d) and, consequently, β3-AR agonism reduced the respiratory exchange ratio (−3.5%; Fig. 2e). This increased EE was accompanied by a marked activation of interscapular BAT (intBAT) as evidenced by reduced intracellular lipid vacuole size in intBAT (−87%; Fig. 3a,b) and reduced weight of BAT pads (approximately −25%; Fig. 1e). UCP1 protein content per area of BAT was increased (+43%; Fig. 3c,d), but not when expressed per total fat pad (Fig. 3e), suggesting mainly the activation of existing BAT. In addition, β3-AR agonism increased browning of WAT (Fig. 3f,g). These data combined thus show that β3-AR agonism effectively activates BAT, enhances EE and increases browning of WAT under atherogenic conditions in *E3L.CETP* mice.

### Activation of BAT increases hepatic VLDL remnant clearance

**Figure 4:**
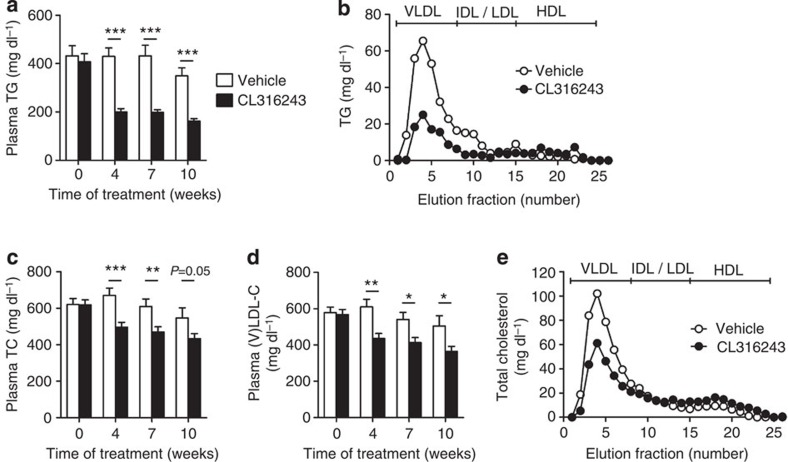
β3-AR agonism ameliorates dyslipidemia in *E3L.CETP* mice. Fasting plasma (**a**) TG, (**c**) TC and (**d**) VLDL-C were measured in WTD-fed *E3L.CETP* at the indicated time points during treatment with the β3-AR agonist CL316243 or vehicle. The distribution of (**b**) TG and (**e**) TC over lipoproteins was determined after 10 weeks of treatment on pooled plasma samples per group. Values are means±s.e.m. (*n*=13–19 per group or pool). *P<0.05, **P<0.01, ***P<0.001 (unpaired two-tailed Student’s *t*-test).

**Figure 5:**
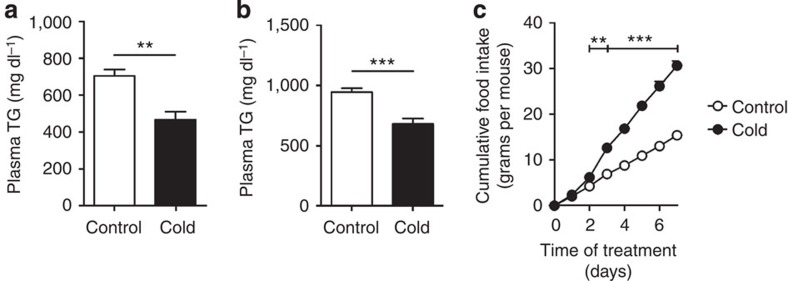
Cold exposure decreases dyslipidemia in *E3L.CETP* mice. *E3L.CETP* mice were fed a WTD and exposed to cold or control temperature for 7 days. Fasted plasma (**a**) TG and (**b**) TC were determined at the end of treatment. (**c**) Mice were individually housed and cumulative food intake was determined during treatment. Values are means±s.e.m. (*n*=8 per group). **P<0.01, ***P<0.001 (unpaired two-tailed Student’s *t*-test).

We next investigated the effect of BAT activation on plasma lipid levels in dyslipidemic *E3L.CETP* mice. BAT activation markedly reduced plasma TG levels throughout the treatment period (approximately −54%; Fig. 4a), caused by a reduction in VLDL-TG (Fig. 4b). Interestingly, BAT activation consistently reduced plasma total cholesterol (TC) levels (approximately −23%; Fig. 4c), also confined to a reduction in the (V)LDL fraction (approximately −27%; Fig. 4d,e), without altering hepatic expression of genes relevant for lipid and lipoprotein metabolism (that is, *Apob*, *CETP*, *Ldlr* and *Mttp*; Supplementary Table 1). This lipid-lowering effect of CL316243-mediated brown fat activation did not differ from cold-induced effects, as cold exposure reduced hyperlipidaemia in *E3L.CETP* mice as well (Fig. 5a,b), despite a marked increase in dietary cholesterol intake in the cold (Fig. 5c).

**Figure 6:**
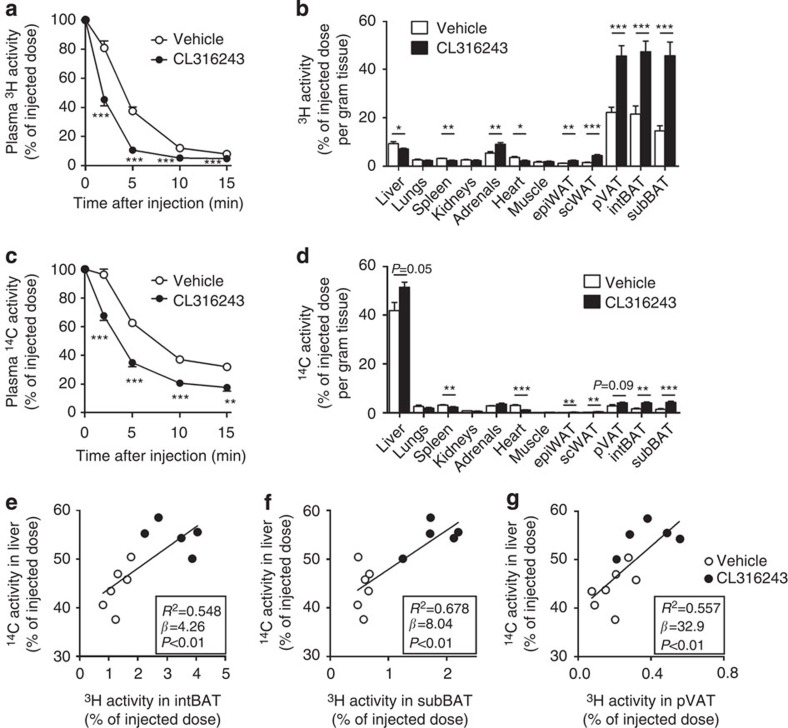
β3-AR agonism increases lipolytic processing and hepatic clearance of lipoproteins in *E3L.CETP* mice. β3-AR agonist CL316243- and vehicle-treated mice were injected with VLDL-like particles, double-labelled with glycerol [^3^H]TO and [^14^C]CO, and (**a**,**c**) clearance from plasma as well as (**b**,**d**) uptake by organs and tissues at 15 min after injection were determined for (**a**,**b**) ^3^H-activity and (**c**,**d**) ^14^C-activity. The hepatic uptake of ^14^C activity was plotted against the uptake of [^3^H]TO-derived activity in (**e**) interscapular brown adipose tissue (intBAT), (**f**) subscapular BAT (subBAT) and (**g**) perivascular adipose tissue (pVAT). Linear regression analyses were performed. Values are means±s.e.m. (*n*=5–7 per group). *P<0.05, **P<0.01, ***P<0.001 (unpaired two-tailed Student’s *t*-test).

We hypothesized that BAT activation reduces VLDL-TG as well as (V)LDL-C, as a result of enhanced uptake of TG-derived FA by BAT. Thus, the lipolytic processing of (V)LDL particles may lead to the formation of smaller cholesterol-enriched lipoprotein remnants, which are prone to be subsequently cleared by the liver. Therefore, we studied the *in vivo* plasma clearance and organ uptake of glycerol tri[^3^H]oleate (triolein, TO)- and [^14^C]cholesteryl oleate (CO)-double-labelled VLDL-mimicking particles (Fig. 6 and Supplementary Fig. 1)^29^ in *E3L.CETP* mice that were treated for 8 days with CL316243, which is compared with the long-term protocol (10 weeks as above) already sufficient to stimulate BAT activity and browning.

In vehicle-treated mice, the plasma decay of [^3^H]TO (Fig. 6a) was faster than that of [^14^C]CO (Fig. 6c; *t*_½_=2.9±0.1 versus 4.7±0.5 min; unpaired two-tailed Student’s *t*-test, *P*<0.01), indicating that lipolytic processing and FA uptake precedes the clearance of core remnants. The uptake of [^3^H]TO-derived activity by the various BAT depots was much higher than by other metabolically active tissues (Fig. 6b). The uptake of ^3^H-activity by BAT depots (Fig. 6b) was much larger than that of ^14^C-activity (Fig. 6d; average ratio ^3^H/^14^C=‘lipolysis index’=11.1±0.9), indicating selective uptake of [^3^H]TO-derived [^3^H]oleate rather than uptake of whole particles. Instead, the delipidated remnants were mainly taken up by the liver (Fig. 6b,d and Supplementary Fig. 1b; lipolysis index=0.22±0.01).

β3-AR agonism markedly accelerated the plasma clearance of [^3^H]TO (*t*_½_=1.5±0.1 versus 2.9±0.1 min; unpaired two-tailed Student’s *t*-test, *P*<0.001; Fig. 6a) and [^14^C]CO (t_½_=3.3±0.3 versus 4.7±0.5 min; Fig. 6c). The accelerated clearance of [^3^H]TO was explained by a pronounced increased uptake of [^3^H]oleate by the various BAT depots (2–3 fold; Fig. 6b and Supplementary Fig. 1a), in accordance with increased BAT activity. The relative increase of [^3^H]TO uptake into BAT is paralleled by [^14^C]CO uptake, indicating that activation of BAT via β3-AR agonism increases both FA and holoparticle uptake pathways to a similar extent. Interestingly, although the specific uptake by the WAT depots was still ∼15-fold lower as compared with the BAT depots, β3-AR agonism also increased the uptake of [^3^H]oleate by these WAT depots (2- to 3-fold), possibly reflecting the activity of beige adipocytes within WAT. Concomitantly, BAT activation increased the uptake of [^14^C]CO by the liver, by ∼25% (Fig. 6d and Supplementary Fig. 1b), indicating accelerated hepatic clearance of core remnants. The remnants that were generated by BAT activation and subsequently taken up by the liver were depleted from TG and more enriched in cholesterol as compared with remnants in vehicle-treated mice as indicated by an ∼30% reduction in lipolysis index (0.15±0.01 versus 0.22±0.01; unpaired two-tailed Student’s, *t*-test *P*<0.01; calculated from Fig. 6b,d).

**Figure 7:**
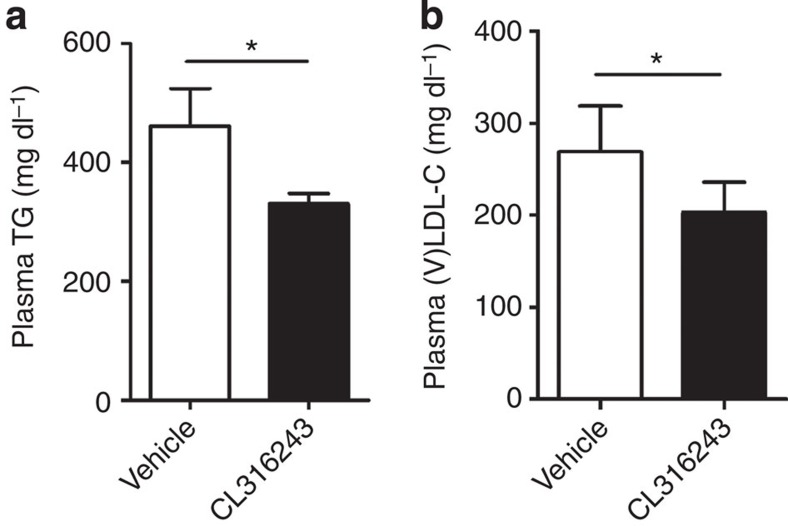
β3-AR agonism reduces dyslipidemia in *E3L* mice in the absence of CETP. *E3L* mice were fed a WTD and treated with vehicle or the β3-AR agonist CL316243. Fasted plasma (**a**) TG and (**b**) (V)LDL-C were determined. Values are means±s.e.m. (*n*=5–7 per group). *P<0.05 (unpaired two-tailed Student’s *t*-test).

Interestingly, additional univariate regression analyses showed that the uptake of TG-derived FA (^3^H activity) by the various BAT depots predicted the uptake of the cholesterol-enriched remnants (^14^C activity) by the liver (Fig. 6e–g). This lipid-lowering effect of β3-AR agonism did not depend on CETP, as CL316243 also reduced plasma lipid levels in *E3L* mice without CETP (Fig. 7). Hence, BAT-mediated lipolytic processing of VLDL promotes the clearance of the corresponding cholesterol-rich remnants by the liver.

### Activation of BAT reduces atherosclerosis development

**Figure 8:**
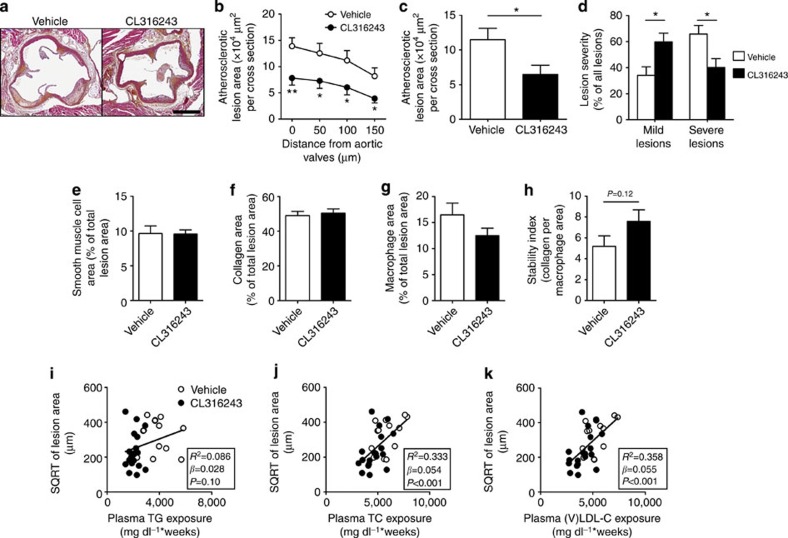
BAT activation reduces atherosclerosis by improving the plasma cholesterol profile. (**a**) Slides of the valve area of the aortic root of β3-AR agonist CL316243- and vehicle-treated *E3L.CETP* mice were stained with haematoxylin–phloxine–saffron and representative pictures are shown. Scale bar, 400 μm. (**b**) Lesion area as a function of distance was determined per mouse, starting from the appearance of open aortic valve leaflets covering 150 μm. (**c**) The mean atherosclerotic lesion area was determined from the four cross-sections from **b** and (**d**) lesions were categorized according to lesion severity. (**e**) The smooth muscle cell, (**f**) collagen and (**g**) macrophage content of the lesions were determined, and (**h**) the stability index (collagen/macrophage content of the lesions) was calculated. The SQRT of the atherosclerotic lesion area from **b** was plotted against the plasma (**i**) total TG, (**j**) TC and (**k**) (V)LDL-C exposure during the 10-week treatment period. Linear regression analyses were performed. Values are means±s.e.m. (*n*=13–19 per group). *P<0.05, **P<0.01 (**b**–**h**: unpaired two-tailed Student’s *t*-test).

Next, we studied whether the decreased (V)LDL levels were accompanied by reduced atherosclerosis development. To this end, we determined atherosclerotic lesion area as well as severity and composition of the lesions in the root of the aortic arch after 10 weeks of BAT activation. Indeed, sustained BAT activation by β3-AR agonism markedly reduced atherosclerotic lesion area throughout the aortic root (Fig. 8a,b), resulting in 43% lower mean atherosclerotic lesion area (Fig. 8c). BAT activation clearly reduced the severity of the lesions, as indicated by more mild lesions (that is, type I–III; +75%) and less severe lesions (that is, type IV–V; −38%; Fig. 8d) without significantly affecting atherosclerotic lesion composition (that is, collagen, vascular smooth muscle cell and macrophage content of the lesions; Fig. 8e–g) or the lesion stability index (that is, ratio collagen/macrophage area; Fig. 8h).

To evaluate the contribution of TG versus TC lowering to the reduction in atherosclerosis, univariate regression analyses were performed. To linearize data for analysis, the atherosclerotic lesion area was square root (SQRT) transformed^27^ and plotted against the TG and TC exposure (that is, area under the curve of plasma lipid for the complete treatment period). We found that the SQRT of the lesion area did not correlate with plasma TG exposure (*β*=0.028; *R*^2^=0.086; *P*=0.10; Fig. 8i) but correlated with plasma TC exposure (*β*=0.054; *R*^2^=0.333; *P*<0.001; Fig. 8j). Additional univariate regression analyses showed that the (V)LDL-C exposure specifically predicts the SQRT of the lesion area (*β*=0.055; *R*^2^=0.358; *P*<0.001; Fig. 8k). Taken together, these analyses reveal that a reduction in plasma (V)LDL-C is the main contributor to the anti-atherogenic effect of β3-AR-mediated activation of BAT.

### Anti-atherogenic effect depends on hepatic remnant clearance

**Figure 9:**
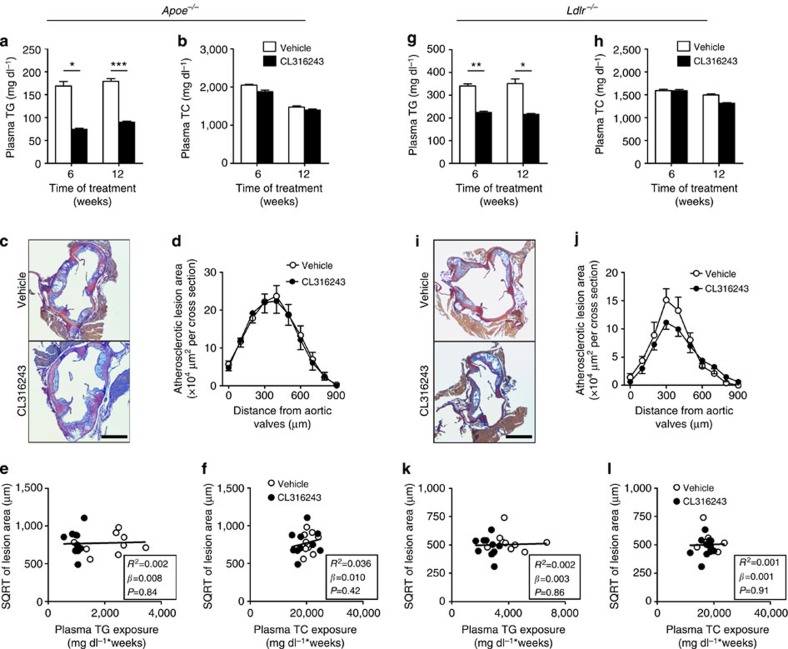
BAT activation in *Apoe*^−/−^ and *Ldlr*^−/−^ mice reduces plasma TG, but not TC nor atherosclerosis. Fasting plasma (**a**) TG and (**b**) TC levels were measured in WTD-fed *Apoe*^−/−^ mice at the indicated time points during treatment with the β3-AR agonist CL316243 or vehicle. (**c**) Slides of the valve area of the aortic root of CL316243- or vehicle-treated *Apoe*^−/−^ mice were stained with modified Van Gieson stain and representative pictures are shown. Scale bar, 400 μm. (**d**) Lesion area as a function of distance was determined per mouse starting from the appearance of open aortic valve leaflets covering 900 μm. The SQRT of the atherosclerotic lesion area from **d** in *Apoe*^−/−^ mice was plotted against the plasma (**e**) total TG and (**f**) TC exposure during the 12-week treatment period. Linear regression analyses were performed. Fasting plasma (**g**) TG and (**h**) TC levels were measured in WTD-fed *Ldlr*^−/−^ mice at the indicated time points during treatment with the β3-AR agonist CL316243 or vehicle. (**i**) Slides of the valve area of the aortic root of CL316243- or vehicle-treated *Ldlr*^−/−^ mice were stained with modified Van Gieson stain and representative pictures are shown. Scale bar, 400 μm. (**j**) Lesion area as a function of distance was determined per mouse starting from the appearance of open aortic valve leaflets covering 900 μm. The SQRT of the atherosclerotic lesion area from **j** in *Ldlr*^−/−^ mice was plotted against the plasma (**k**) total TG and (**l**) TC exposure during the 12-week treatment period. Linear regression analyses were performed. Values are means±s.e.m. (*n*=8–11 per group or pool). *P<0.05, **P<0.01, ***P<0.001 (**a**,**b**,**d**,**g**,**h**,**j**: unpaired two-tailed Student’s *t*-test).

As we observed increased hepatic uptake of lipoprotein remnants after CL316243-induced BAT activation (Fig. 6d), we evaluated the role of the apoE–LDLR axis, the main hepatic route for clearance of TRL remnants^21^. Cold adaptation^20^ and CL316243 treatment (data not shown) increased food intake in both *Apoe*^−/−^ and *Ldlr*^−/−^ mice. Therefore, to match dietary cholesterol intake, we studied the effect of β3-AR-mediated BAT activation in WTD-pair-fed *Apoe*^−/−^ mice and *Ldlr*^−/−^ mice. Similar as in *E3L.CETP* mice, activation of BAT reduced body weight, WAT pad size and plasma TG levels (Fig. 9a and Supplementary Fig. 2a–c). However, activation of BAT did neither reduce plasma TC and (V)LDL-C levels (Fig. 9b and Supplementary Fig. 2d), change hepatic gene expression (Supplementary Table 1), nor did it reduce atherosclerosis development (Fig. 9c,d) in *Apoe*^−/−^ mice. As expected, the SQRT of the lesion area did not correlate with either total plasma TG exposure or plasma TC exposure during the study (Fig. 9e,f).

As the LDLR is the main hepatic clearance receptor for apoE-containing lipoprotein remnants^21^, we next investigated the role of the LDLR for the (V)LDL-C-reducing effect of BAT activation. Similar as in *E3L.CETP* and *Apoe*^−/−^ mice, activation of BAT reduced body weight, WAT pad size and plasma TG levels in WTD-pair-fed *Ldlr*^−/−^ mice (Fig. 9g and Supplementary Fig. 3a–c). However, activation of BAT in *Ldlr*^−/−^ mice did neither reduce plasma TC and (V)LDL-C levels (Fig. 9h and Supplementary Fig. 3d), change messenger RNA levels of selected hepatic genes (Supplementary Table 1), nor reduce atherosclerosis development (Fig. 9i,j), nor did the SQRT of the lesion area correlate with the total plasma TG and TC exposure during the study (Fig. 9k,l). Taken together, these findings demonstrate that activation of BAT reduces plasma (V)LDL-C and subsequently reduces atherosclerosis development through enhanced LDLR-mediated hepatic clearance of apoE-containing lipoprotein remnants.

## Discussion

Since its rediscovery in human adults in the last decade^2^,^4^,^5^,^6^,^7^,^30^, BAT has been considered a promising therapeutic target for obesity and associated metabolic disorders. The anti-obesity potential of BAT has been irrefutably proven in murine studies^18^,^19^,^31^ and shown in human studies^4^,^30^,^32^,^33^. However, in light of the study by Dong *et al*.^20^, the effect of BAT activation on cholesterol metabolism and atherosclerosis development remained controversial to date. Here we present evidence that, in principle, BAT activation is beneficial and not deleterious for plasma cholesterol metabolism and atherosclerosis development. Using *E3L.CETP* and *E3L* mice, we demonstrate that BAT-mediated local lipolysis of TG-rich lipoproteins stimulated hepatic clearance of lipoprotein remnants via a pathway involving the apoE–LDLR pathway. As a result, BAT activation reduced atherosclerotic lesion size and severity in *E3L.CETP* mice but not in mice lacking either apoE or LDLR.

Our study indicates that the ability of the liver to clear apoE-enriched lipoprotein remnants via the LDLR is a prerequisite for the anti-atherogenic potential of BAT activation. According to this view, it is not surprising that Dong *et al*.^20^ recently observed that BAT activation by cold exposure in *Apoe*^−/−^ and *Ldlr*^−/−^ mice actually increased plasma (V)LDL-C levels and atherosclerosis. Likewise, in these hyperlipidemic mouse models prolonged BAT activation results in lipoprotein remnant levels in plasma exceeding the hepatic clearance capacity. In fact, Dong *et al*.^20^ did show that activation of BAT by cold in normolipidemic wild-type C57Bl/6 mice actually decreased plasma TC and (V)LDL-C levels, which is in full accordance with a functional hepatic apoE–LDLR clearance route of lipoprotein remnants in wild-type mice. It is also reasonable that in mice lacking UCP1 in addition to apoE or LDLR, the formation of remnants is impaired, thereby apparently paradoxically protecting these mice from atherosclerosis^20^. As we observe that both CL316243 treatment (Fig. 4) and cold exposure (Fig. 5a,b) reduce hyperlipidaemia in *E3L.CETP* mice our results provide proof for the concept that in principle BAT activation has beneficial effects on plasma lipids.

In this study, we used TRL-mimicking particles as models for TRLs. We previously showed that these particles rapidly acquire an array of exchangeable apolipoproteins from serum, including apoE, apoCs, apoAIV, apoAI, apoAII and apoD^29^,^34^, and that the hepatic uptake of their core remnants is mediated by apoE^34^. In fact, the *in-vivo* kinetics of [^3^H]cholesteryl oleate-labelled TRL-mimicking particles^34^ are very similar to those of [^3^H]vitamin A-labelled native chylomicrons^35^, with similar clearance rate from plasma (*t*_½_ ∼2 min) and uptake by the liver (∼65%–75% after 30 min). Lactoferrin reduced the uptake by the liver of both TRL-mimicking particles and chylomicrons by ∼75%^35^ and liver cell distribution studies confirmed that hepatocytes accounted for ∼75% of the total uptake by the liver^35^. Taken together, it is likely to be that the present findings with TRL-mimicking particles can be translated to endogenous TLRs.

Our work sets the foundation for future studies that investigate the anti-atherogenic potential of BAT in humans, especially since a recent study demonstrated that β3-AR agonism activates BAT and increases EE in humans^36^. As the discovery that functional BAT is present and active in human adults was made only in the last decade^2^,^4^,^5^,^6^,^7^,^30^, recent studies have focused on the physiological relevance of BAT for humans. The concept of BAT being involved in energy metabolism and being a target for obesity was appreciated some decades ago based on experimental studies in rodents^37^,^38^. More recently, its role in human energy metabolism and obesity development became apparent as well, from observations that BAT activity inversely correlates with obesity^39^, and that cold acclimation recruits BAT^40^ and lowers fat mass^33^. In addition, South Asians, who have a high susceptibility to metabolic disorders, have decreased EE associated with decreased BAT volume and activity compared with white Caucasians, although it should be noted that body composition is different between these ethnicities^41^. Importantly, BAT activation by means of cold acclimation also improved cholesterol metabolism in human patients with hypercholesterolaemia^42^, underscoring the potential of BAT activation as a potential anti-atherogenic treatment in humans.

In conclusion, our data demonstrate that activation of BAT lowers atherogenic lipoprotein levels and protects against atherosclerosis development. Moreover, we show the importance of a functional hepatic apoE–LDLR clearance pathway, rendering *Apoe*^−/−^ or *Ldlr*^−/−^ mice inappropriate models for studying the beneficial effects of BAT modulation on plasma cholesterol metabolism and atherosclerosis development. We propose that BAT activation, resulting in accelerated generation of lipoprotein remnants, should preferentially be combined with strategies that increase hepatic LDLR expression, including statins and/or proprotein convertase subtilisin/kexin type 9 blockers, thus fully unravelling the therapeutic potential of BAT for atherosclerosis prevention and treatment.

## Methods

### Mice and animal procedures

Female *E3L* were cross-bred with mice expressing human CETP under control of its natural flanking regions, to generate heterozygous *E3L.CETP* mice^25^. Female *E3L.CETP* and *E3L* mice were 10–12 weeks of age and individually housed under standard conditions with a 12-h light–dark cycle, free access to food and water, and 22 °C room temperature. Mice were fed a WTD (HopeFarms, Woerden, The Netherlands) supplemented with 0.1% cholesterol and treated with the β3-AR agonist CL316243 (Tocris Bioscience Bristol, UK; 3 × 20 μg per mouse per week; subcutaneous) or vehicle (PBS) for 10 weeks. Food intake and body weight, as well as total body fat and lean mass, were monitored by EchoMRI-100 (EchoMRI, Houston, Texas) during the studies.

Male *Apoe*^−/−^ and *Ldlr*^−/−^ mice (Jackson Laboratory, Bar Harbor, ME) were individually housed and fed a WTD supplemented with 0.2% cholesterol with or without CL316243 (0.001% w/w). As CL316243 treatment of *Apoe*^−/−^ and *Ldlr*^−/−^ mice increased food intake, these mice were pair-fed to their respective controls after onset of effect. All animal experiments were approved by the Ethics Committee on Animal Care and Experimentation of the Leiden University Medical Center or The Animal Welfare Committee of University Medical Center Hamburg-Eppendorf.

### Indirect calorimetry

Indirect calorimetry was performed in fully automatic metabolic cages (LabMaster System, TSE Systems, Bad Homburg, Germany) during the ninth week of treatment. After 1 day of acclimatization, O_2_ consumption, CO_2_ production and caloric intake were measured for 3 consecutive days^42^. Total EE was estimated from the VO_2_ and resting energy requirement. Carbohydrate oxidation was calculated using the formula ((4.585*VCO_2_)−(3.226*VO_2_))*4, in which the 4 represents the conversion from mass per time unit to kcal per time unit^43^. Similarly, fat oxidation was calculated using the formula ((1.695*VO_2_)−(1.701*VCO))*9. Values were normalized to lean mass. Physical activity was monitored using infrared sensor frames. The first 12 h directly after injection with CL316243 or vehicle (‘Day of treatment’) were analysed and compared with the same 12-h period 24 h later (‘Day after treatment’).

### Cold exposure

Mice were individually housed with *ad libitum* access to WTD and water during exposure to 4 °C in a cold room with a 12-h light–dark cycle. After 7 days of cold exposure 4 h-fasted plasma lipids were determined as described below.

### *In-vivo* plasma decay and organ uptake of VLDL-like particles

VLDL-like TG-rich emulsion particles (80 nm) labelled with glycerol tri[^3^H]oleate (TO) and [^14^C]CO were prepared and characterized as described previously^29^. Briefly, [^3^H]TO (100 μCi) and [^14^C]CO (10 μCi) tracers were added to a mixture of TO (70 mg), egg yolk phosphatidylcholine (22.7 mg), lysophosphatidylcholine (2.3 mg), CO (3.0 mg) and cholesterol (2.0 mg). Particles were prepared from this mixture at 54 °C using a Soniprep 150 (MSE Scientific Instruments, UK) at 10 μm output. The emulsion was fractionated by consecutive density gradient ultracentrifugation steps in a Beckman SW 40 Ti rotor. After centrifugation (27 min, 20,000 r.p.m., 20 °C) the top fraction containing chylomicron-like particles with an average size of 150 nm was discarded. After a subsequent centrifugation step (27 min, 40,000 r.p.m., 20 °C) VLDL-like particles with an average size of 80 nm were isolated, stored at 4 °C under argon and used within 5 days.

After 8 days of treatment with the β3-AR agonist CL316243 (20 μg per mouse per day) mice were fasted for 4 h and injected (*t*=0) via the tail vein with the emulsion particles (1.0 mg TG per mouse in 200 μl PBS). Blood samples were taken from the tail vein at 2, 5, 10 and 15 min after injection, to determine the plasma decay of [^3^H]TO and [^14^C]CO. Plasma volumes were calculated as 0.04706 × body weight (g) as determined from ^125^I-labelled BSA clearance studies as described^44^. After 15 min, mice were killed by cervical dislocation and perfused with ice-cold heparin solution (0.1% v/v in PBS) via the heart, to remove blood from the organs and tissues. Subsequently, organs and tissues were isolated, dissolved overnight at 56 °C in Tissue Solubilizer (Amersham Biosciences, Rosendaal, The Netherlands) and quantified for ^3^H and ^14^C activity. Uptake of [^3^H]TO- and [^14^C]CO-derived radioactivity by the organs and tissues was expressed per gram wet tissue weight or calculated as dose per organ after correction for organ weight.

### Plasma parameters and lipoprotein profiles

Blood was collected from the tail vein of 4-h fasted mice into EDTA- (*E3L.CETP* and *E3L* mice) or lithium-heparin-coated tubes (*Apoe*^−/−^ and *Ldlr*^−/−^ mice). Tubes were placed on ice, centrifuged and plasma was isolated and assayed for TG and TC by using commercially available enzymatic kits from Roche Diagnostics (Mannheim, Germany). Plasma (V)LDL-C levels were calculated by extraction of HDL-C from TC levels. HDL-C levels were determined by precipitating apoB-containing lipoproteins from plasma by addition of 20% polyethylene glycol in 200 mM glycine buffer with pH 10 and TC was measured in the supernatant as described above. The distribution of TG and cholesterol over lipoproteins was determined in pooled plasma by fast-performance liquid chromatography on a Superose 6 column (GE Healthcare, Piscataway, NJ).

### Hepatic gene expression analysis

Total RNA was extracted from snap-frozen livers using Tripure RNA isolation reagent (Roche, Mijdrecht, The Netherlands), according to the manufacturer’s instructions. Complementary DNA for quantitative reverse transcriptase–PCR was produced by reverse transcription of total RNA using Moloney Murine Leukemia Virus Reverse Transcriptase (Promega, Leiden, The Netherlands). mRNA expression was normalized to *β2-microglobulin* and *36b4* mRNA content and expressed as fold change compared with control mice. The primer sequences used are listed in Supplementary Table 2.

### Necropsy and histology of BAT and WAT

At the end of the study, mice were anaesthetized by intraperitoneal injection of acepromazine (6.25 mg kg^−1^; Alfasan, Woerden, The Netherlands), midazolam (0.25 mg kg^−1^; Roche, Mijdrecht, The Netherlands) and fentanyl (0.31 mg kg^−1^; Janssen-Cilag, Tilburg, The Netherlands), bled and killed by cervical dislocation or transcardial blood withdrawal. The blood circulation was perfused with ice-cold heparin solution (0.1% v/v in PBS), and organs and tissues were weighed and collected for further analyses. Epididymal WAT and intBAT were removed and preserved in phosphate-buffered 4% formaldehyde and embedded in paraffin. Haematoxylin–eosin staining was performed using standard protocols. Intracellular lipid droplet size in WAT and percentage of lipid-droplet-positive area in BAT were quantified using Image J software (version 1.47).

For UCP1 staining, 5-μm sections were deparafinnized in xylene, rehydrated in ethanol and treated with 3% H_2_O_2_ (Sigma-Aldrich, Zwijndrecht, The Netherlands) in absolute methanol for 30 min, to block endogenous peroxidase activity. Sections were immersed in citrate buffer (10 mM, pH 6) and boiled for 10 min. Slides were blocked with 1.3% normal goat serum (in PBS) and incubated overnight at 4 °C with rabbit monoclonal anti-UCP1 antibody (Abcam, Cambridge, UK; 1:400 in 1.3% normal goat serum). Subsequently, sections were incubated for 60 min with biotinylated goat α-rabbit secondary antibody (Vector Labs, Burlingame, CA) diluted in 1.3% normal goat serum. Immunostaining was amplified using Vector Laboratories Elite ABC kit (Vector Labs) and the immunoperoxidase complex was visualized with Nova Red (Vector Labs). Counterstaining was performed with Mayer’s haematoxylin (1:4). Expression of UCP1 was quantified using Image J software (version 1.47) and expressed per area and per total fat pad.

### Atherosclerosis quantification

Hearts were collected and fixated in phosphate-buffered 4% formaldehyde, embedded in paraffin and cross-sectioned (5 μm) perpendicular to the axis of the aorta throughout the aortic root area, starting from the appearance of open aortic valve leaflets. Per mouse, four sections with 50-μm intervals were used for atherosclerosis measurements. Sections were stained with haematoxylin–phloxine–saffron for histological analysis. For *Apoe*^−/−^ and *Ldlr*^−/−^ mice, paraffin-embedded hearts were cross-sectioned (10-μm) and stained with modified Van Gieson stain. Per mouse, three consecutive cross-sections per 100-μm intervals were used for atherosclerosis measurements, starting at the appearance of open aortic valve leaflets. Lesions were categorized for lesion severity according to the guidelines of the American Heart Association adapted for mice^45^. Various types of lesions were discerned: mild lesions (types 1–3) and severe lesions (types 4–5). Lesion area was determined using Image J Software (version 1.47). Quantification of smooth muscle cell actin was performed using antibody M0851 (Dako, Carpinteria, CA) and DAKO envision anti-mouse (Vector Laboratories Inc., Burlingame, CA). Rat monoclonal anti-MAC-3 antibody (BD Pharmingen, San Diego, CA) and Vector Impress anti-rat (Vector Laboratories Inc.) were used to quantify macrophage area. Immunostainings were amplified using Vector Laboratories Elite ABC kit (Vector Laboratories Inc.) and the immunoperoxidase complex was visualized with Nova Red (Vector Laboratories Inc.). Sirius red (Chroma, Stuttgart, Germany) was used to stain for collagen. The stability index was calculated by dividing the collagen by the macrophage area.

### Statistical analyses

Statistical analyses between genotypes were assessed using the unpaired two-tailed Student’s *t*-test. Univariate regression analyses were performed to test for significant correlations between uptake of ^14^C activity in the liver and ^3^H activity in intBAT, and between atherosclerotic lesion area and plasma lipid exposures during the study. Multiple regression analysis was performed to predict the contribution of plasma TG and TC exposures during the study to the atherosclerotic lesion area. The SQRT of the lesion area was taken to linearize the relationship with the plasma lipid exposures. Data are presented as mean±s.e.m., unless indicated otherwise. Differences at a probability level (*P*) of 0.05 were considered statistically significant. SPSS 20.0 for Windows (SPSS, Chicago, IL) was used for statistical analyses.

## Author contributions

J.F.P.B, A.B., J.H. and P.C.N.R. designed the experiments, with the help from M.R.B. and P.P.S.J.K. J.F.P.B., M.R.B., P.P.S.J.K., A.B., C.S., A.W., S.K., G.H., I.M.M., C. John, C. Jung, N.V., L.P.J.B., P.L.S.M.G. and L.S. performed experiments and analysed data. J.F.P.B., M.R.B., P.P.S.J.K., A.B., G.H., P.L.S.M.G., J.H. and P.C.N.R. interpreted data. J.D.E, P.S.H., L.M.H. and L.D. provided intellectual contributions throughout the project. J.F.P.B., M.R.B., P.P.S.J., A.B., J.H. and P.C.N.R. wrote the manuscript. All authors discussed the results and commented on the manuscript. J.H. and P.C.N.R. were responsible for the overall supervision of the study.

## Additional information

**How to cite this article:** Berbée, J. F. P. *et al*. Brown fat activation reduces hypercholesterolaemia and protects from atherosclerosis development. *Nat. Commun.* 6:6356 doi: 10.1038/ncomms7356 (2015).

## Supplementary Material

Supplementary Information Supplementary Figures 1-3 and Supplementary Tables 1-2
