# Supplementary material for: Brown fat activation reduces hypercholesterolaemia and protects from atherosclerosis development
Source: Nat Commun. 2015 Mar 10;6:6356. doi: 10.1038/ncomms7356 (PMC4366535; doi:10.1038/ncomms7356)
Supplement: Supplementary Information — Supplementary Figures 1-3 and Supplementary Tables 1-2 [file ncomms7356-s1.pdf]

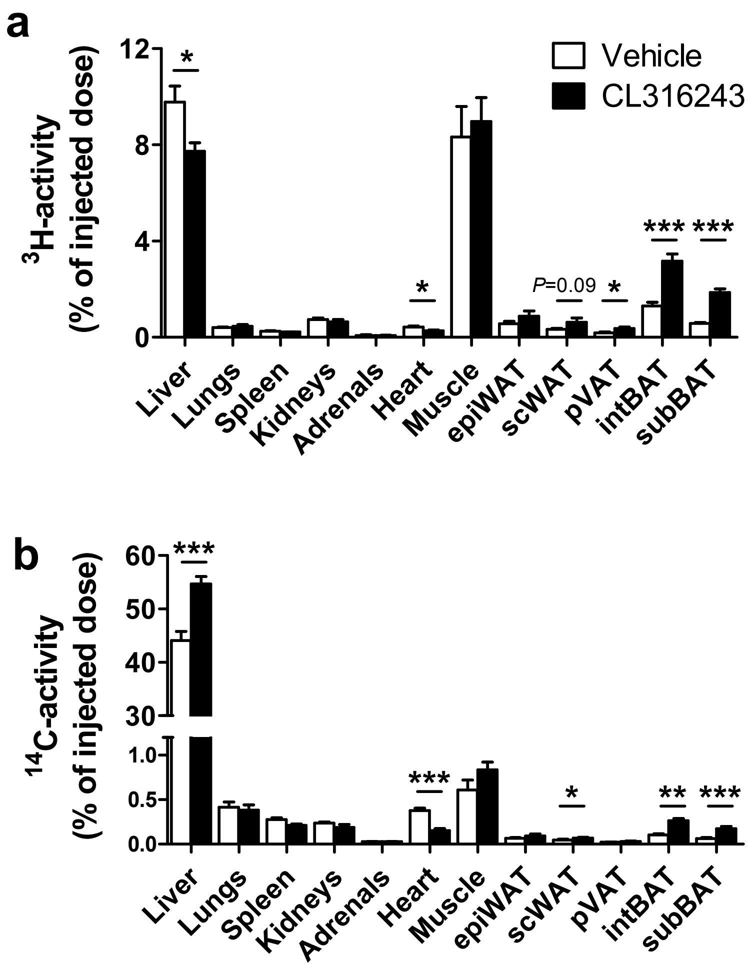

Supplementary Fig. 1. BAT activation enhances lipolytic processing and hepatic clearance of lipoproteins.

E3L.CETP mice were fed a Western-type diet and treated with vehicle or the β3-AR agonist CL316243 for 8 consecutive days. Fasted mice were injected with glycerol tri[^{3}H]oleate- and [^{14}C]cholesteryl oleate-labeled VLDL-mimicking emulsion particles and after 15 min uptake of (**a**) ^{3}H-label and (**b**) ^{14}C-label was determined in the indicated tissues. Data are expressed as percentage ^{3}H- or ^{14}C-activity of the injected dose per total tissue, with an exception for subcutaneous WAT (scWAT; determined as uptake in 2 representative fat pads) and perivascular adipose tissue (pVAT; determined as uptake in a representative part surrounding the aorta). EpiWAT, epididymal WAT; subBAT, subscapular BAT; intBAT, interscapular BAT. Values are means ± S.E.M. (n=5-7 per group). *P<0.05, **P<0.01, ***P<0.001 (unpaired two-tailed Student’s t-test).

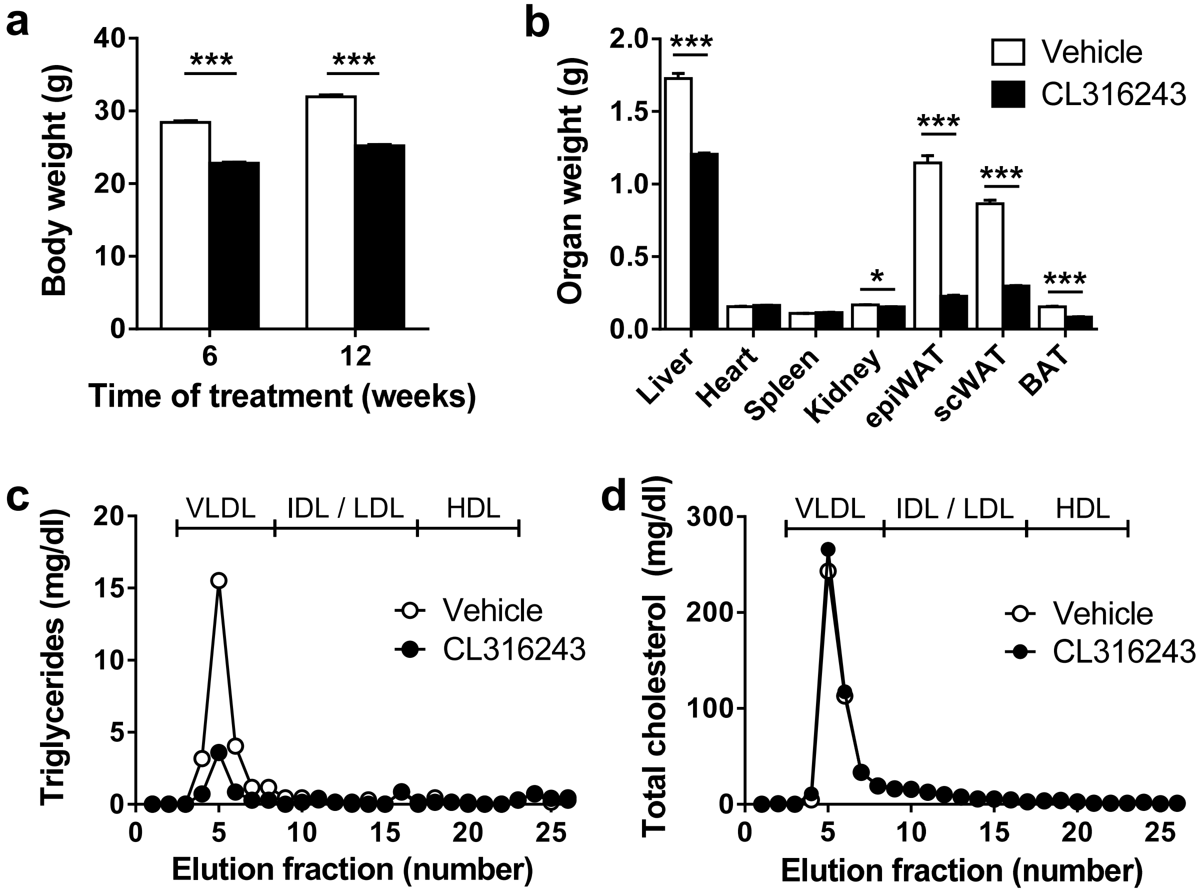

Supplementary Fig. 2. BAT activation in *Apoe*$^{-/-}$ mice reduces fat mass and plasma triglycerides, but not total cholesterol.

Apoe$^{-/-}$ mice were fed a Western-type diet and treated with vehicle or the β3-AR agonist CL316243, pair-fed to the vehicle-treated group, during 12 weeks. (**a**) Body weight was determined after 6 and 12 weeks of treatment. (**b**) Organ weight was determined after necropsy at week 12 (B). The distribution of (**c**) triglycerides and (**d**) cholesterol over lipoproteins was determined at week 12 in fasted plasma samples that were pooled per group. Values are means ± S.E.M. (n=9-10 per group). **P*<0.05, ****P*<0.001 (unpaired two-tailed Student's t-test).

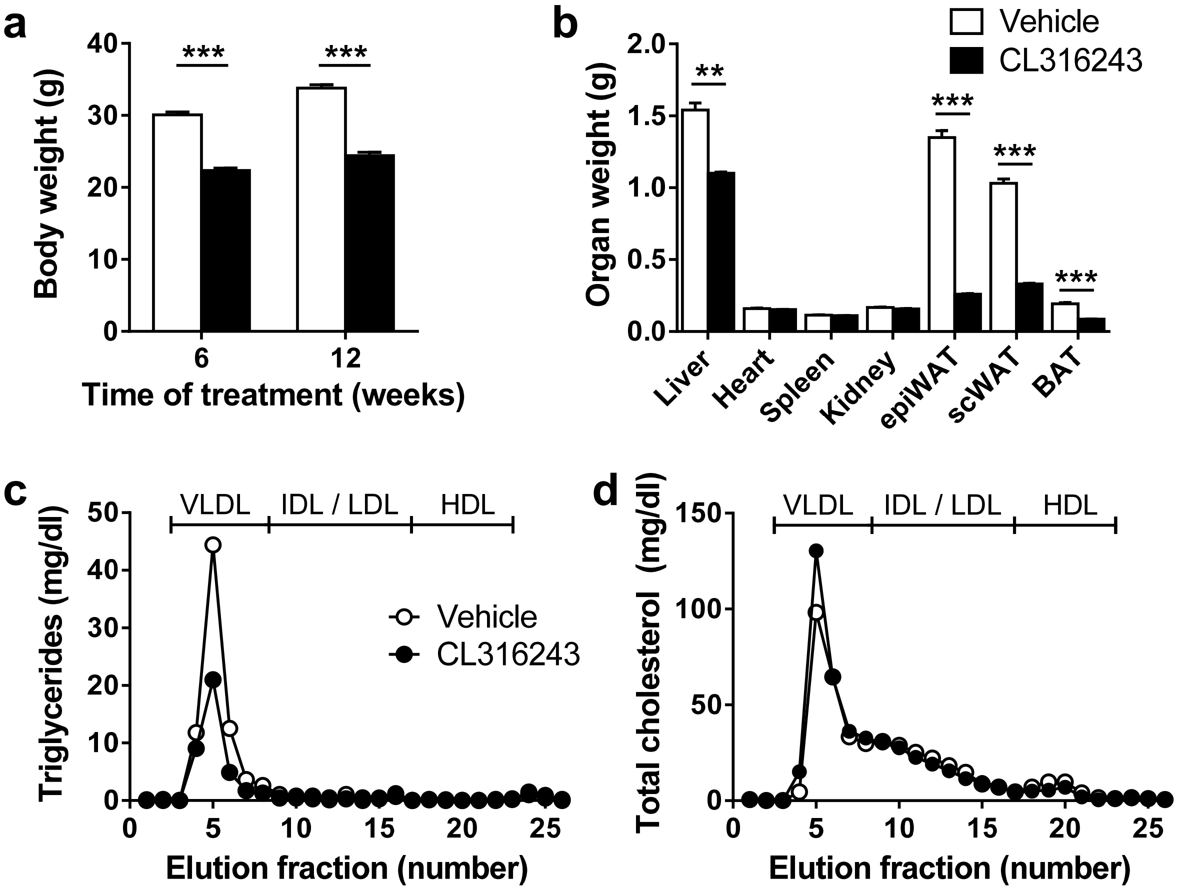

Supplementary Fig. 3. BAT activation in *Ldlr*$^{-/-}$ mice reduces fat mass and plasma triglycerides, but not total cholesterol.

Ldlr$^{-/-}$ mice were fed a Western-type diet and treated with vehicle or the β3-AR agonist CL316243, pair-fed to the vehicle-treated group, during 12 weeks. (**a**) Body weight was determined after 6 and 12 weeks of treatment. (**b**) Organ weight was determined after necropsy at week 12. The distribution of (**c**) triglycerides and (**d**) cholesterol over lipoproteins was determined at week 12 in fasted plasma samples that were pooled per group. Values are means ± S.E.M. (n=8-11 per group). $^{**}P<0.01$, $^{***}P<0.001$ (unpaired two-tailed Student's t-test).

Supplementary Table 1. β3-AR agonism does not alter hepatic expression of genes involved in lipid and lipoprotein metabolism[1-3]

	E3L.CETP		*Apoe*$^{-/-}$		*Ldlr*$^{-/-}$	
**Gene**	**Vehicle**	**CL316243**	**Vehicle**	**CL316243**	**Vehicle**	**CL316243**
Apob	1.00 ± 0.06	1.02 ± 0.07	1.00 ± 0.02	1.09 ± 0.03	1.00 ± 0.03	1.10 ± 0.04
CETP	1.00 ± 0.17	1.09 ± 0.18	n.d.	n.d.	n.d.	n.d.
Ldlr	1.00 ± 0.08	0.87 ± 0.02	1.00 ± 0.10	0.82 ± 0.08	n.d.	n.d.
Mttp	1.00 ± 0.06	1.19 ± 0.21	1.00 ± 0.05	0.96 ± 0.05	1.00 ± 0.07	0.98 ± 0.06

[1] mRNA expression is expressed as fold change as compared to vehicle mice.

[2] *Apob*, apolipoprotein B; *CETP*, cholesteryl ester transfer protein; *Ldlr,* low-density lipoprotein receptor; *Mttp,* microsomal triglyceride transfer protein.

[3] Statistical differences were assessed with unpaired two-tailed Student's t-test. (n=5-8). N.d., not determined.

Supplementary Table 2. List of primer sequences qRT-PCR[1]

Gene	Forward primer	Reverse Primer
36b4	GGACCCGAGAAGACCTCCTT	GCACATCACTCAGAATTTCAATGG
Apob	GCCCATTGTGGACAAGTTGATC	CCAGGACTTGGAGGTCTTGGA
β2-microglobulin	TGACCGGCTTGTATGCTATC	CAGTGTGAGCCAGGATATAG
CETP	CAGATCAGCCACTTGTCCAT	CAGCTGTGTGTTGATCTGGA
Ldlr	GCATCAGCTTGGACAAGGTGT	GGGAACAGCCACCATTGTTG
Mttp	CTCTTGGCAGTGCTTTTTCTCT	GAGCTTGTATAGCCGCTCATT

[1] *Apob*, apolipoprotein B; *CETP*, cholesteryl ester transfer protein; *Ldlr*, low-density lipoprotein receptor; *Mttp,* microsomal triglyceride transfer protein.
